# Prehistoric women’s manual labor exceeded that of athletes through the first 5500 years of farming in Central Europe

**DOI:** 10.1126/sciadv.aao3893

**Published:** 2017-11-29

**Authors:** Alison A. Macintosh, Ron Pinhasi, Jay T. Stock

**Affiliations:** 1 PAVE (Phenotypic Adaptability, Variation and Evolution) Research Group, Department of Archaeology, University of Cambridge, Cambridge CB2 3DZ, UK.; 2 Department of Anthropology, University of Vienna, Althanstrasse 14, 1090 Vienna, Austria.; 3 Department of Anthropology, Western University, London, Ontario N6A 3K7, Canada.

## Abstract

The intensification of agriculture is often associated with declining mobility and bone strength through time, although women often exhibit less pronounced trends than men. For example, previous studies of prehistoric Central European agriculturalists (~5300 calibrated years BC to 850 AD) demonstrated a significant reduction in tibial rigidity among men, whereas women were characterized by low tibial rigidity, little temporal change, and high variability. Because of the potential for sex-specific skeletal responses to mechanical loading and a lack of modern comparative data, women’s activity in prehistory remains difficult to interpret. This study compares humeral and tibial cross-sectional rigidity, shape, and interlimb loading among prehistoric Central European women agriculturalists and living European women of known behavior (athletes and controls). Prehistoric female tibial rigidity at all time periods was highly variable, but differed little from living sedentary women on average, and was significantly lower than that of living runners and football players. However, humeral rigidity exceeded that of living athletes for the first ~5500 years of farming, with loading intensity biased heavily toward the upper limb. Interlimb strength proportions among Neolithic, Bronze Age, and Iron Age women were most similar to those of living semi-elite rowers. These results suggest that, in contrast to men, rigorous manual labor was a more important component of prehistoric women’s behavior than was terrestrial mobility through thousands of years of European agriculture, at levels far exceeding those of modern women.

## Teaser

The advent of farming was associated with thousands of years of manual labor for women that exceeded that of living athletes.

## INTRODUCTION

Over the past 30 years, sexual dimorphism has been documented in anthropological studies examining temporal trends in bone strength associated with the intensification of agriculture and the development of sedentism (*1*–*5*). Trends among women are often less pronounced [although see the studies of Bridges *et al.* (*6*) and Ruff *et al.* (*7*)] or follow different patterns than those of men who conform more consistently to expectations about changes in mobility and behavior on the basis of archeological evidence (*5*, *8*–*10*). As a result, it is the adaptive responses and behavioral trends of men that have historically received the most attention (*11*–*14*), whereas sexual dimorphism in the response of bone properties to mechanical loading remains poorly understood and comparative limb bone mechanical property data from women of known activity are completely lacking. Thus, the typically less dramatic patterns of adaptation and lower mean limb bone strength among women have not been explored in sufficient resolution. The extent to which less pronounced temporal trends among women might reflect high internal variability in female behavior, thereby reducing interpopulation variation in morphology, or might reflect actual sexual divisions of labor, biological differences in bone plasticity to loading, or other factors remains unclear.

Analyses of cultural and biological interactions among mid-Holocene Central European agriculturalists have identified a pronounced diachronic pattern of decline in tibial loading among men, suggesting a corresponding decline in mobility. In Central Europe, in the period from the Early Neolithic through to the Late Iron Age [~5300 calibrated years (cal) BC to 100 AD], on average, male tibial diaphyses became significantly straighter and, in cross section, smaller in area, less rigid, and more circular (*9*, *10*). Because of the strong and consistent relationships that have been established between mechanical loading and cross-sectional limb bone size, rigidity, and shape among living humans (*11*, *12*, *15*), these morphological changes provide convincing evidence of changing loading, and thus likely changing mobility among prehistoric men, through time. The consistent regional trend in tibial morphology noted among Central European men is supported by the larger European trends documented by Ruff *et al.* (*7*) from the Upper Paleolithic (33,000 to 11,000 BP) to very recent (≥1900 AD) time periods. A comparison of prehistoric data for Central European males with those of living male athletes and control subjects further supports a relationship between tibial cross-sectional geometry and the advent of sedentism: Mean tibial rigidity and shape ratios among Neolithic men were similar to those of male cross-country runners and had declined to the level of sedentary control subjects by approximately 385 cal BC (Late Iron Age), where mean values remained into the Medieval period (*9*, *12*).

In contrast, prehistoric women from the same cemeteries exhibited less pronounced lower limb trends or none at all, making the interpretation of female mobility challenging. The only significant diachronic change among Central European women appeared to be in the predominance of anteroposterior (A-P) loading through time, where Medieval women had significantly straighter tibial diaphyses that were more circular in cross section than their earliest farming counterparts in the Neolithic period (*9*, *10*). This finding is supported by the gradual decline in A-P strength of the tibia, as documented by Ruff and colleagues (*7*), among women across Europe in the Holocene. Despite fewer significant trends in tibial rigidity and shape among Central European women relative to contemporaneous men, manual activities appear to have changed substantially more among women than men during this time (*16*). Upper limb bone mechanical properties were initially variable and right-lateralized in the Neolithic period among these Central European women and became highly symmetrical and homogeneous in the Bronze Age, a change that was attributed to the increasing predominance of bimanual cereal processing using saddle querns in the region [see also the study by Sládek (*17*)]. Thus, manual labor may have been a more intensive component of behavior than was terrestrial mobility among mid-Holocene Central European female agriculturalists, who consistently exhibited significantly lower values in all virtually lower limb properties and fewer significant diachronic trends than men (*9*, *10*).

However, the evidence of sex differences in norms of reaction of bone to mechanical loading makes it inappropriate to interpret female behavior in the past via the direct comparison of mechanical properties to those of males. For example, among modern tennis players, side-to-side differences document substantially more responsiveness to mechanical loading in the male relative to the female skeleton (*18*, *19*). Further, sex differences in bone strength parameters do not appear to be due to larger average body size among males because they persist even when these differences are controlled for (*20*). The surface-specific osteogenic effects of sex hormones (*21*), sex differences in the secretion of growth hormone and insulin-like growth factors and in their receptors (*22*), and sex differences in growth trajectory (*23*) may all result in the greater ability of male bone to respond to loading in a mechanically advantageous manner than female bone. Experimental data from laboratory rats support trends documented among humans: Järvinen *and colleagues* (*24*) found clear sex-specific differences in the sensitivity of the femoral neck to loading. They found that exercised male rats exhibited much greater responsiveness to mechanical loading than did females and an increased capacity for geometrical adaptation, whereas female rats developed denser bones relative to mechanical demand. This extra accumulation of bone mineral in females is documented among human women at puberty in response to elevated estrogen secretion, allowing for the storage of calcium in preparation for pregnancy, lactation, and/or menopause (*25*), but potentially limiting the adaptive response to loading in comparison to males (*24*). Thus, additional factors may moderate the typical functional and energetic influences on bone mass and distribution among women. However, there is currently a lack of comparative limb bone mechanical property data from women of known activity with which to compare prehistoric female data and a resultant lack of understanding of sexual dimorphism in the response of bone to mechanical loading. A direct understanding of the relationship between mechanical loading and long-bone cross-sectional geometry among women is crucial to understand not only women’s behavior through time but also sociocultural change through the development of agrarian and production economies worldwide.

Here, we investigate temporal trends in upper and lower limb bone cross-sectional geometric (CSG) properties and interlimb strength proportions among prehistoric women spanning the first ~6150 years of agriculture in Central Europe (~5300 cal BC to 850 AD) in relation to a comparative group of living European women of known behavior. Interlimb strength proportions between the humerus and the tibia were used to characterize the relative importance of manual labor versus terrestrial mobility among agricultural women. Although tibio-humeral strength proportions have never been used in the analysis of behavioral differences among human populations, femoro-humeral structural proportions have proven useful in this regard (*14*). Further, the utility of interlimb strength proportions for distinguishing broad differences in locomotor behaviors (arboreality and terrestrial mobility) among primate species and early hominins has been well established (*26*–*29*).

Size-standardized humeral and tibial polar second moments of area (*J*), cross-sectional shape (*I*_max_/*I*_min_), and interlimb strength proportions are compared between Neolithic, Bronze Age, Iron Age, and Medieval women and living female athletes, as well as recreationally active control subjects as a reference group of low-impact loading. Athletes were included from three sports that load the limbs with differing intensity and directionality: (i) endurance running, high lower limb loading based on ground reaction force and unidirectional loading trajectories; (ii) football (soccer), high lower limb loading based on ground reaction force and multidirectional loading trajectories; and (iii) rowing, higher repetitive upper limb loading based primarily on joint contact forces and unidirectional loading trajectories. This comparative data set was used to explore the following questions: (i) To what extent can the apparent homogeneity in interpopulation variation in female tibial morphology among early agricultural women be explained by high internal variability? (ii) Were Central European prehistoric farming women more mobile than living sedentary women? (iii) Among prehistoric Central European females, was manual labor a more rigorous behavioral component of agricultural intensification than terrestrial mobility?

## RESULTS

### Upper and lower limb solid CSG properties

**Fig. 1:**
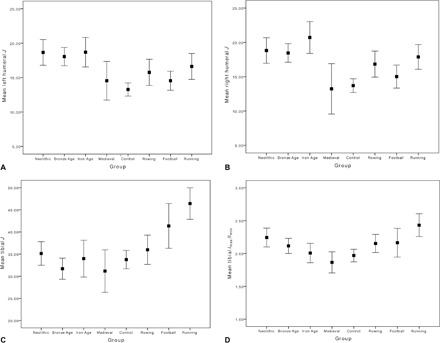
Humeral and tibial CSG properties among the prehistoric and modern groups. (**A**) Polar second moment of area (*J*), left humerus, 35% section location (*n* = 157). (**B**) Polar second moment of area (*J*), right humerus, 35% section location (*n* = 151). (**C**) Polar second moment of area (*J*), tibia, 50% section location (*n* = 173). (**D**) *I*_max_/*I*_min_, tibia, 50% section location (*n* = 178). Data are given as means ± 95% confidence interval (CI). Summary statistics by group are given in Table 1, and means were compared using one-way ANOVA.

Summary statistics for all female solid-section CSG properties by group are presented in Table 1, and the results of one-way analyses of variance (ANOVAs) by group are given in Table 2. Prehistoric agricultural labor among Central European women appears to have been dominated by upper limb loading until at least the Late Iron Age (~100 AD), at levels much higher than those seen among most living women (Fig. 1, A and B). In the left humerus, mean bending/torsional rigidity (polar second moment of area, *J*) was significantly higher among Neolithic, Bronze Age, and Iron Age women than rowers, football players, and controls. In the right humerus, mean *J* was significantly higher among Neolithic, Bronze Age, and Iron Age women than football players and control subjects. Iron Age women also had significantly higher mean humeral rigidity than Medieval women. In contrast, mean midshaft tibial *J* (Fig. 1C) was significantly lower among women in all prehistoric time periods than it was among living endurance runners (*P* < 0.001 for all). Mean tibial *J* among football players also significantly exceeded the particularly low values among Bronze Age and Medieval women.

**Table 1:** Solid-section female limb bone summary statistics by group. All properties derived from solid cross sections. Values are given as means (SD). Medieval humeral values were obtained from three-dimensional (3D) laser scans. All other prehistoric humeral values were obtained from silicone molds. Coefficients of variation are calculated as (SD/mean) × 100. –, variation not examined due to small sample size; CV, coefficient of variation.

		**Tibia**						
	***n***	**Size-standardized *J***			***n***	***I*_max_/*I*_min_**		
		**Mean**	**Range**	**CV (%)**		**Mean**	**Range**	**CV (%)**
**Prehistoric**								
Neolithic	31	35.14 (7.26)	22.92–53.41	20.66	34	2.24 (0.41)	1.43–3.13	18.30
Bronze Age	30	31.70 (6.39)	19.43–46.59	20.16	32	2.12 (0.32)	1.55–3.03	15.09
Iron Age	17	33.98 (8.09)	22.38–49.08	23.81	13	2.01 (0.25)	1.52–2.41	12.44
Medieval	11	31.16 (7.15)	23.15–47.92	22.95	11	1.87 (0.24)	1.35–2.10	12.83
**Modern**								
Running	18	46.41 (7.16)	33.08–59.18	15.42	18	2.43 (0.35)	1.88–3.18	14.40
Football	11	41.36 (7.53)	30.31–56.23	18.21	11	2.16 (0.33)	1.59–2.78	15.28
Rowing	16	35.98 (6.65)	24.88–47.00	18.48	16	2.15 (0.28)	1.67–2.78	13.02
Controls	37	33.76 (6.26)	23.26–45.52	18.54	37	1.97 (0.29)	1.46–2.75	14.72
		**Humerus**						
	***n***	**Left size–standardized *J***			***n***	**Right size–standardized *J***		
		**Mean**	**Range**	**CV (%)**		**Mean**	**Range**	**CV (%)**
**Prehistoric**								
Neolithic	29	18.65 (4.92)	11.42–27.89	26.38	27	18.82 (4.70)	12.16–28.67	24.97
Bronze Age	27	18.05 (3.37)	12.52–25.55	18.67	28	18.46 (3.49)	12.93–25.82	18.91
Iron Age	17	18.69 (4.17)	12.71–26.07	22.31	14	20.71 (4.01)	14.53–25.38	19.36
Medieval	5	14.54 (2.26)	11.56–16.75	—	7	13.23 (3.97)	7.32–18.25	—
**Modern**								
Running	17	16.62 (3.67)	11.01–26.55	22.08	16	17.89 (3.38)	13.06–25.49	18.89
Football	11	14.54 (2.05)	10.44–17.50	14.10	10	15.02 (2.35)	10.40–18.52	15.65
Rowing	17	14.62* (3.71)	12.35–27.21	23.56	16	16.83 (3.56)	11.98–26.23	21.15
Controls	35	13.27 (2.77)	7.99–20.12	20.87	35	13.70 (2.90)	8.79–20.83	21.17

*Value given is median (SD) due to non-normal distribution (mean value, 15.75).

**Table 2:** Results of post hoc (ANOVA) comparisons of bone CSG properties and linear regression standardized residuals. All ANOVAs are significant to *P* < 0.001. Bold indicates significant post hoc *P* values. All values of *J* were size-standardized. Run, endurance runners; FB, football players; Row, rowers; Neo, neolithic; BA, Bronze Age; IA, Iron Age; Med, Medieval period; SR Hum:Tib, standardized residuals of regression of humeral and tibial *J*.

**Bone variable**	**Neo**	**BA**	**IA**	**Med**	**Run**	**FB**	**Row**
Tibial *I*_max_/*I*_min_							
BA	0.959						
IA	0.523	1.0					
Med	**<0.027**	0.545	1.0				
Run	0.734	**<0.033**	**<0.012**	**<0.001**			
FB	1.0	1.0	0.999	0.588	0.595		
Row	1.0	1.0	0.999	0.453	0.259	1.0	
Control	**<0.014**	0.823	1.0	1.0	**<0.001**	0.897	0.750
Tibial *J*														
BA	0.774						
IA	1.0	1.0					
Med	0.945	1.0	1.0				
Run	**<0.001**	**<0.001**	**<0.001**	**<0.001**			
FB	0.269	**<0.003**	0.166	**<0.019**	0.801		
Row	1.0	0.664	1.0	0.86	**<0.001**	0.701	
Control	1.0	0.999	1.0	1.0	**<0.001**	**<0.046**	1.0
Left humeral J*							
BA	0.980						
IA	0.973	0.709					
Med	0.093	**<0.024**	0.078				
Run	0.311	0.138	0.196	0.170			
FB	**<0.008**	**<0.004**	**<0.008**	0.955	0.115		
Row	**<0.026**	**<0.013**	**<0.02**	0.724	0.310	0.689	
Control	**<0.001**	**<0.001**	**<0.001**	0.297	**<0.001**	0.160	**<0.038**
Right humeral *J*														
BA	1.0						
IA	0.873	0.633					
Med	0.108	0.128	**<0.024**				
Run	0.994	0.999	0.458	0.225			
FB	**<0.05**	**<0.036**	**<0.005**	0.949	0.224		
Row	0.768	0.818	0.143	0.488	0.988	0.765	
Control	**<0.001**	**<0.001**	**<0.001**	1.0	**<0.005**	0.808	0.081
SR Hum:Tib *J*														
BA	0.247						
IA	1.0	0.654					
Med^†^	1.0	0.181	0.992				
Run	0.072	**<0.001**	**<0.037**	0.145			
FB	**<0.003**	**<0.001**	**<0.002**	**<0.017**	0.983		
Row	1.0	0.461	0.993	1.0	0.572	0.163	
Control	**<0.009**	**<0.001**	**<0.009**	0.079	1.0	0.704	0.550

*Kruskal-Wallis test was performed due to non-normality of distribution in one of the groups, with two-tailed Mann-Whitney post hoc comparisons.

†Small sample size (*n* = 6).

Despite having significantly lower mean tibial *J* than endurance runners, Neolithic women did not differ significantly from them in tibial *I*_max_/*I*_min_ (cross-sectional shape; Fig. 1D). These relatively high mean *I*_max_/*I*_min_ values among Neolithic women (more elliptical cross sections expanded in the A-P direction) significantly exceeded those of Medieval women and living control subjects. Bronze Age, Iron Age, and Medieval women and living control subjects all had significantly lower mean *I*_max_/*I*_min_ (more circular cross sections) than did endurance runners.

**Fig. 2:**
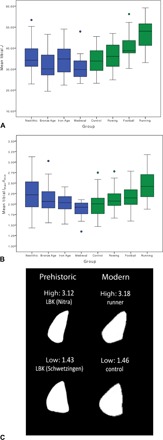
Variation in tibial *J* and *I*_max_/*I*_min_ among prehistoric and living women. (**A**) Box plot of midshaft tibial *J* values showing the median, upper and lower quartiles, and interquartile range by group (*n* = 173). (**B**) Box plot of midshaft tibial *I*_max_/*I*_min_ values showing the median, upper and lower quartiles, and interquartile range by group (*n* = 178). (**C**) Midshaft tibial solid-section images showing the variation in *I*_max_/*I*_min_ values among Early Neolithic LBK women and living women. Summary statistics by group are given in Table 1.

Variation within prehistoric time periods exceeded that documented within living female groups (see Table 1), particularly in tibial *J* (Fig. 2A). Behavioral variation among Early Neolithic women, all belonging to the Linearbandkeramik (LBK) culture, was notably high; LBK variability in left humeral rigidity exceeded 26%, and tibial shape variation encompassed the entire range of living women pooled, with the exception of one individual (Fig. 2, B and C). In contrast, variability in upper limb loading intensity was lower in the Bronze Age (18.5 to 19%), reflecting a narrower range of loading than was documented among living rowers (21.15 to 23.56%), a sport involving repetitive and homogeneous upper limb movement.

### Relative upper and lower limb loading intensity

Results of one-way ANOVAs on standardized residuals for mean raw combined left and right humeral *J* relative to raw tibial *J* are presented in Table 2. All prehistoric women had high relative upper limb loading when compared to living women. This distribution did not differ significantly from that of rowers, and it differed to the greatest extent from that of football players, among whom relative loading is biased heavily toward the lower limb. As a result, women at all time periods had significantly more strengthened humeri relative to tibiae than football players, whereas Neolithic, Bronze Age, and Iron Age women also differed significantly from control subjects. In particular, Bronze Age and Iron Age women exhibited pronounced relative interlimb differences in loading, biased to the upper limb to the greatest extent, significantly so relative to endurance runners among both time periods.

### Sexual dimorphism in true CSG properties

Table 3 provides summary statistics for size-standardized peripheral quantitative computed tomography (pQCT)–derived true CSG property means (derived from the original pQCT images incorporating both periosteal and endosteal contours) from the tibial midshaft among living male and female endurance runners and control subjects. All male data are taken from means published in the study by Shaw and Stock (*12*). Means among women are consistently lower than among males for bending/torsional rigidity, maximum bending rigidity, minimum bending rigidity, shape ratio, cortical bone area, and percent cortical bone area, regardless of the intensity with which individuals are loading their limbs.

**Table 3:** Size-standardized pQCT-derived true CSG property means from the tibial midshaft among living males and females. All male data are taken from Shaw and Stock (*15*), and all male data were size-standardized following the methods outlined in Shaw and Stock (*15*). *I*_max_, maximum second moment of area, quantifies maximum bending/torsional rigidity; *I*_min_, minimum second moment of area, quantifies minimum bending/torsional rigidity; CA, cortical bone area; %CA, percent cortical bone area (relative to total subperiosteal area).

	***n***	**Property**					
		***J***	***I*_max_**	***I*_min_**	***I*_max_/*I*_min_**	**CA**	**%CA**
**Runners**							
Males	15	50.13 (7.81)	35.93 (4.23)	14.20 (2.86)	2.60 (0.50)	6.20 (0.51)	79.50 (2.93)
Females	18	44.07 (6.45)	31.50 (5.17)	12.57 (1.89)	2.53 (0.38)	5.91 (0.51)	77.89 (4.75)
**Controls**							
Males	20	38.10 (7.91)	26.46 (4.23)	11.64 (1.20)	2.28 (0.28)	5.22 (0.58)	74.60 (5.01)
Females	37	31.11 (5.68)	20.74 (4.00)	10.37 (2.04)	2.02 (0.31)	4.72 (0.60)	72.71 (4.12)

## DISCUSSION

**Fig. 3:**
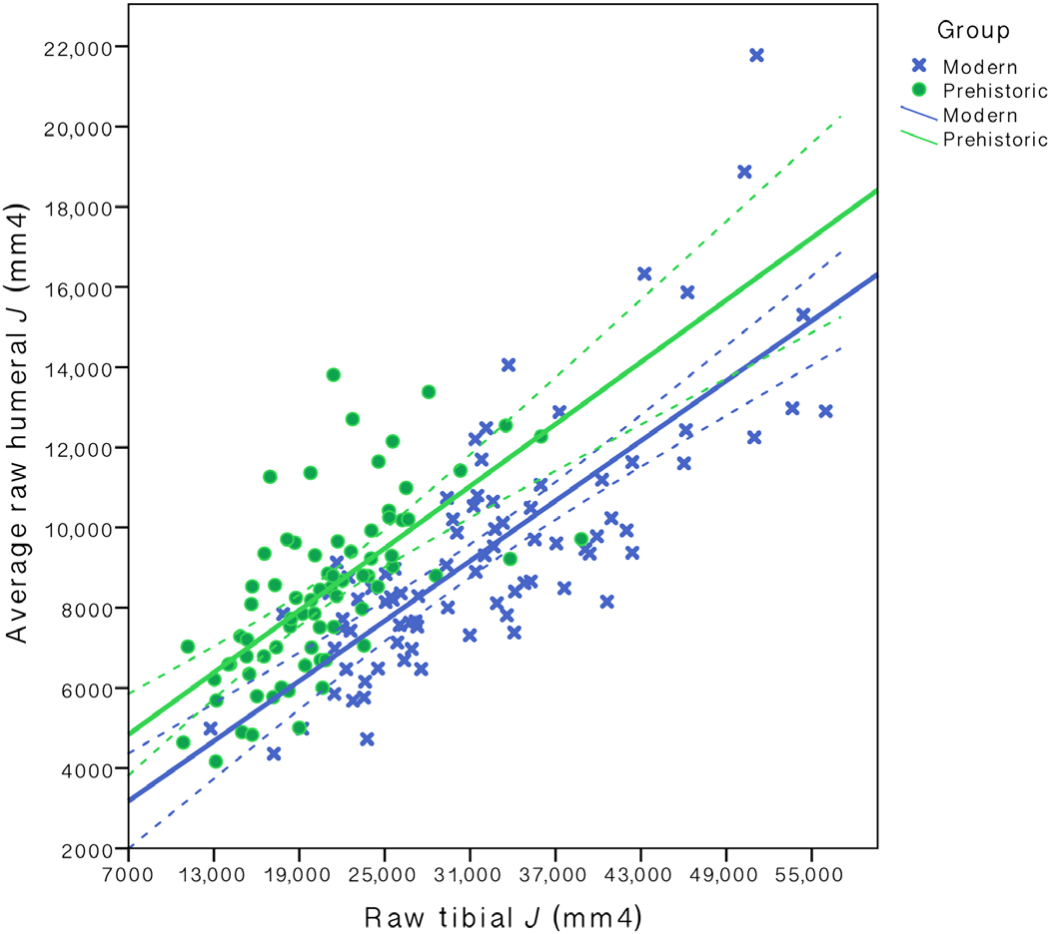
Interlimb strength proportions among prehistoric and agricultural women. Scatterplot of humeral versus tibial raw *J* (mm^4^) among prehistoric agricultural women (green) and living women (blue) (*n* = 159). Solid and dashed lines represent the lines of best fit and 95% CI, respectively. Group comparisons were performed using one-way ANOVA on standardized residuals from regressions of average raw humeral *J* to raw tibial *J*.

The current study identified very high levels of upper limb loading among most prehistoric agricultural women when compared to both living female athletes and controls. The distribution of loading between the upper and lower limbs was biased heavily toward the former among prehistoric women, to a greater extent than among all living women, including semi-elite rowers (see Fig. 3). Thus, the intensification of agriculture was associated with very high levels of manual labor relative to terrestrial mobility for women, and changes in the female behavioral repertoire through time are less heavily characterized by declining terrestrial mobility than among contemporaneous men (*9*, *10*).

All mean prehistoric female values for tibial rigidity were well below those documented among living female endurance runners, and it is not likely that average terrestrial mobility among most agricultural women involved high ground reaction forces. Rather, mean tibial rigidity among women spanning the ~6150 years (5300 cal BC to 850 AD) between the Early Neolithic and Early Medieval periods remained very similar to that of living recreationally active control subjects (low impact) and varsity rowers (no ground impact but high muscle magnitudes) when adjusted for body size. However, there was substantial overlap in tibial rigidity between prehistoric and living women, even endurance runners: Some women within all time periods exhibit size-standardized tibial *J* values at or above the mean for endurance runners, yet others fall well below the mean for controls. Among Early Neolithic LBK women alone, tibial rigidity ranged from below the lowest control to the highest portion of endurance runners, and, on average, LBK women did not differ significantly from football players in tibial rigidity. Consistently high variability in lower limb mechanical properties within populations reduces the ability to detect variation in mobility between groups. Further, this internal variability may explain why female trends in tibial rigidity did not generally conform to expectations based on mobility, such as those of their male counterparts.

Many of the Neolithic LBK cemeteries included in the analyses here have been well studied, providing substantial evidence of female behavioral variation across many aspects of life. Analyses of strontium isotope residues from sites including Vedrovice, Schwetzingen, and Nitra provide evidence of a patrilocal kinship system, with women being more likely than men either to have originated from or obtained their subsistence from areas outside of the preferred LBK loess soil areas (*30*). These sex differences in residential mobility patterns among the LBK may contribute to more variable signatures of mobility in women than in men. There is little evidence to suggest that frequent short-term fluctuation in logistic mobility might be contributing to high variation in habitual activities among LBK women. The LBK exhibit considerable cultural homogeneity throughout their Central European distribution, with little temporal or geographic variation in settlement type and location, house construction, or characteristic polished adze-axe stone tools and pottery (*31*).

It is difficult to specifically determine the extent to which LBK women participated in agricultural or livestock-related activities that may have contributed to their moderate but variable tibial loading. However, in many modern agricultural groups, women are responsible for the majority of subsistence tasks related to gathering and hoe agriculture (*32*), as well those related to domestic animal care (*33*). Collecting fodder for domesticated animals accounts for up to 5 hours per day of work for women in some modern intensive agriculturalist societies (*34*), and other time-consuming tasks include fetching water for livestock, caring for young animals, milking, and processing milk, meat, hides, and wool (*35*). These tasks involve variable degrees of upper and lower limb loading but overall do not likely require high mobility levels or substantial high-impact lower limb loading. In the Early Neolithic of Central Europe, subsistence activities involved the intensive cultivation of cereals, including emmer, einkorn, spelt, and club wheats as well as barley and millet, and LBK groups predominantly tended cattle as well as pigs, sheep, and goats (*31*). The economic participation of LBK women in livestock-related activities, tilling, planting, and harvesting crops, likely with digging sticks, hoes, and flint sickles inserted into wooden handles (*31*), as well as grinding the grain once harvested, was likely considerable.

The excellent preservation of material culture, including ceramic and pottery vessels, figurines, polished stone tools, bone tools and artifacts, grinding stones, and ovens (*31*, *36*), at many Early Neolithic LBK cemeteries, particularly Vedrovice, provides evidence of a large range of food and object production and processing activities that were being performed as part of daily life at these settlements. Because LBK women are most often buried with pottery, it is likely that they were heavily involved in the production and use of these objects. Further evidence of female involvement in production activities is found at Nitra and Vedrovice, where 25% of individuals (mostly women) show evidence of manipulative tooth wear (*37*). Among modern subsistence agriculturalists, food processing tends to be a predominantly female activity (*38*), and ethnographic observations note that grinding using a saddle quern can burden women with an average of approximately 5 hours a day of manual labor (*39*). The processing of cereals with a saddle quern, the technology available in the Neolithic and Bronze Age, is also relatively inefficient: Sládek and colleagues (*40*) found that grinding grain with a saddle quern required four times more time and two times more muscle activity per kilogram of grain than did grinding with the rotary quern, which was introduced in the Iron Age. Among the LBK, saddle querns were the main tool used for cereal processing. It is probable that upper limb loading associated in part with grain grinding is contributing to substantial relative humeral strengthening among Neolithic LBK women on average.

**Fig. 4:**
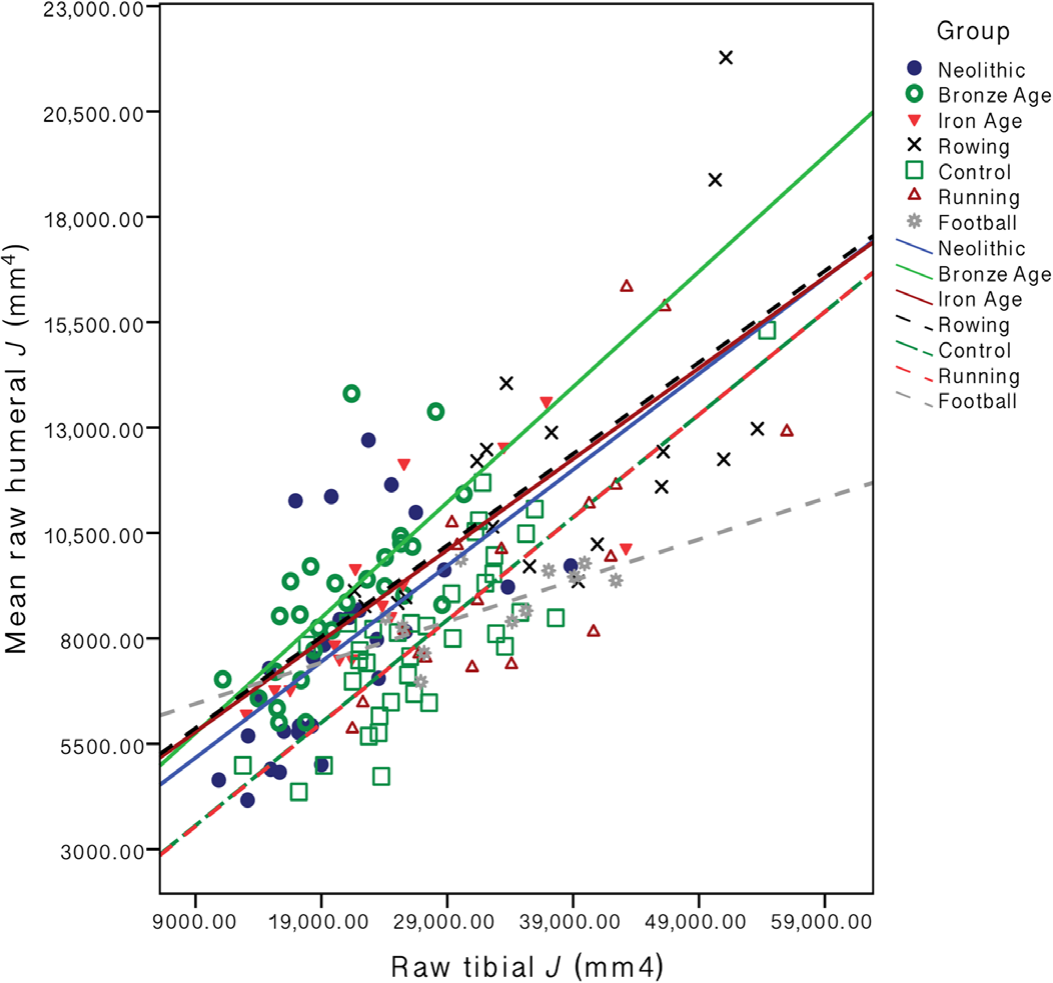
Interlimb strength proportions among prehistoric and agricultural women by group. Scatterplot of humeral versus tibial raw polar second moments of area (*J*; mm^4^) among prehistoric agricultural women and living women by time period and sport (*n* = 159). Solid lines of best fit represent prehistoric populations, and dashed lines of best fit represent living groups.

This intensive manual labor among women prior to mechanization clearly exceeds much of what would be required of the living women in this sample in their day-to-day lives. As a result, relative limb loading among women spanning the Early Neolithic through Late Iron Age in Central Europe (5300 cal BC to 100 AD) is biased heavily onto the upper limb. The distribution of loading between the upper and lower limbs among Early Neolithic LBK women most closely matched the pattern documented among rowers (see Fig. 4); there is no significant difference between LBK women and living rowers in right humeral rigidity, and they have very similar mean midshaft tibial rigidity (35.14 and 35.98, respectively) and shape ratios (2.24 and 2.15, respectively). In the upper limb, rowing exerts significant joint contact forces (forces experienced by the bone/cartilage, including from muscle force) across the elbow and shoulder, and tension forces with the oar handle often exceed the rower’s body weight (*41*). Upper limb loading in rowing is also extremely repetitive: Women rowers in this study trained up to 21 hours per week (up to ~190 km), and they have been rowing for an average of 7 years (4 to 13 years; see table S2). Despite the lack of weight bearing or vertical ground reaction force exerted on the lower limb in rowing, the sport exerts joint contact forces across the ankle, knee, and hip and loads the tibia predominantly in an A-P direction. Further, the powerful muscular co-contractions of the drive phase produce high-peak muscle tension and knee joint contact forces that exceed six times the rower’s body weight (~4100 N) (*41*). These joint contact forces on the lower limb are actually much higher than those experienced during walking (three times body weight) (*42*) or low-impact sports, such as cycling (two times body weight) (*43*). The intensity, directionality, and interlimb distribution of loading exerted by the habitual behaviors of LBK women are most comparable to that of living semi-elite rowers, many of whom have represented their countries at World Rowing Championships, World Rowing U23 Championships, World Junior Championships, and World and European University Championships.

Bronze Age women exhibited lower average tibial rigidity than control subjects but higher average humeral rigidity than all living female groups. This pattern of interlimb loading, heavily biased toward the upper limb, differs most substantially from that of football players, where loading is heavily biased toward the lower limb (see Fig. 4). Football is the only sport included in which participants reported virtually no history of upper limb loading, combined with an average of more than 12 years of high-impact lower limb loading initiated an average of 4 years prior to menarche (see table S1). The mean tibial rigidity of football players exceeds that of all prehistoric means, significantly so relative to these Bronze Age women, while simultaneously, their mean humeral rigidity is significantly lower in both upper limbs. Thus, the relative distribution of loading produced by Bronze Age female habitual behaviors appears to be broadly similar in the extent of interlimb differences as football players but with the reverse pattern; in particular, habitual activities in the Bronze Age were likely completely dominated by upper limb loading relative to lower limb loading.

These results suggest that the behaviors of Central European women in the Bronze Age were dominated by intensive and repetitive manual labor, such as grain grinding, combined with the lowest tibial loading of the prehistoric groups examined. It is likely that food processing remained largely a female activity in the Bronze Age, and saddle querns were still the dominant technology for grinding grain in the Early Bronze Age of Central Europe (2300 to 1500 cal BC). Substantial social stratification at this time, the changing importance of women’s domestic and farming activities with the intensification of agriculture, the shift from hoes and digging sticks to the plow (*32*, *33*, *44*), the emergence of dairying, textile, and metallurgical industries, and the increasing importance of production tasks associated with glass, salt, bone, leather, and antler (*31*, *32*) may have meant that, by the Early Bronze Age, Central European women spent a larger proportion of their time engaged in relatively stationary activities that repetitively and intensively loaded the upper limbs.

Relative workload between men and women in modern agropastoralist societies around the world is variable (*45*), particularly relative to foraging societies, because it is highly dependent on context and ecology. Similar regional variation in the sexual division of labor is possible among prehistoric agropastoralist communities outside of Central Europe [for example, see the studies by Ruff *et al.* (*1*) and Stock and Pfeiffer (*4*)]. However, in the Middle Neolithic (6000 to 5500 BP, uncalibrated) of Western Liguria in Italy (*3*, *5*, *46*), a marked sexual division of labor was documented, with evidence of both low mobility levels among women and the performance of very symmetrical upper limb loading, attributed to the performance of bimanual cereal processing. Similarly, among European women overall (*17*), humeral structural properties shift most with the introduction of agriculture and quern technologies in the Neolithic and subsequent periods, whereas those of men change most prior to the Neolithic, in response to changes in hunting technology and behavior (*17*). In the Americas, women exhibit greater increases in humeral strength than men alongside the intensification of native seed crops (*6*) and of horticultural activities related to surplus food production (*47*), suggesting heavier involvement in these agricultural activities among women than men.

The current study highlights the importance of female comparative data and a female-specific context for the interpretation of female behavior in the past. By interpreting prehistoric human behavior relative to women of known behavioral repertoires, this study has documented thousands of years of very high manual labor among agricultural women in the mid-Holocene of Central Europe. Mean humeral rigidity exceeded all living female means until the Late Iron Age, and loading was biased heavily toward the upper relative to lower limb until the Late Iron Age/Early Medieval period, when it redistributed to a more characteristically modern female pattern. Prehistoric women were also more variable than living women; often, a single time period contained individuals encompassing the entire range of values documented among the entire group of living women ranging from sedentary controls to ultramarathon runners, particularly among the earliest prehistoric populations. This is suggestive of the performance of a wide range of behaviors by early agricultural women in Central Europe and may explain the homogeneity in between-population variation in tibial morphology in females.

If female behavior is interpreted solely through relative differences in limb bone CSG between the sexes, then it is likely that we are underestimating loading intensity among women in the past. Comparison of mean CSG properties between the sexes among living endurance runners and control subjects supports experimental findings by others that male bone responds to loading in a more mechanically advantageous manner than female bone (*18*, *19*, *24*). Male endurance runners had more cortical bone that was distributed with greater anteroposterior expansion and higher average, maximum, and minimum bending/torsional rigidity than did female runners. The same pattern was also true for recreationally active control subjects. Thus, not all components of sexual dimorphism in limb bone CSG among prehistoric males and females can be attributed to actual behavioral differences; some influence of norms of reaction and the surface-specific effects of androgens and estrogen on cortical bone may be influencing the adaptive capacity of bone to loading.

The biological basis of bone morphology is clearly complex, affected by an interplay of genetic and environmental factors that vary in relative importance throughout the skeleton. Important environmental influences include a combination of metabolic stress from factors such as malnutrition, poor health, and physiological stress (*48*) and from mechanical loading and its timing (*49*). There is some evidence of metabolic stress in the earliest stages of farming among these women (*50*) that improved through time, whereas the living women in the study were all healthy and had no history of major medical conditions, eating disorders, immobility, or medications known to affect bone. If dietary and health status affected the cortical thickness or endosteal contour of prehistoric women, then that would be undetectable using solid-section CSG properties derived solely from the periosteal contour and could be contributing slightly to the magnitude of differences between prehistoric and living women. However, for the reconstruction of past loading patterns, solid-section properties provide very accurate estimates of true CSG properties (*51*), particularly at the section locations used in this study. As a result, the impact of higher metabolic stress among prehistoric women on CSG property estimates would likely be minimal.

Differences between prehistoric and living women in the timing at which mechanical loading was initiated during growth could also be affecting the magnitude of differences in CSG property estimates. Loading initiated during growth/adolescence is particularly important for mechanical strength (*49*); although the age at which habitual behaviors were initiated among prehistoric women is unclear, there is some evidence among Neolithic German LBK populations that physical activity and subsistence specialization began from a young age. Juveniles appear to have been more often participating in livestock herding than cultivation (*52*), a specialization that may have contributed to some young LBK boys (~2 to 7 years of age) at Stuttgart-Mühlhausen spending large amounts of time away from the site in their late childhood and early youth (*53*). Physical activity among living controls was low at all ages, whereas 91% of football players began their sport prior to menarche and most other athletes reported some prior history of participation in physical activity during childhood and adolescence. However, the comparability of premenarcheal physical activity levels between prehistoric and these living women is unknown.

Further, there is some suggestion that hormonal contraceptive use may impact bone geometry parameters [for example, the study by Hartard *et al.* (*54*)], with current or past use reported in 73% of living women included in this study. It is unclear whether hormonal contraceptive use is impacting the magnitude of difference between living and prehistoric women identified in the current study, and more work is needed in this area before any conclusions can be drawn. In addition, the fibula was not assessed in the current study because its structural properties do not respond as strongly to loading as those of the tibia among living humans (*55*), and its loading environment is less well understood. However, the fibula appears important in mediolateral loading in living humans (*56*), and the bone is a good indicator of positional behaviors among hominoids (*57*), so its consideration may have provided additional information on loading and mobility patterns among Central European women.

Although these factors promote caution when interpreting differences in bone CSG properties among prehistoric and living European women, having comparative data from living women clearly affords a more accurate means of interpreting female behavior in the past than do male data. A broader understanding of bone variation and norms of reaction among women is essential because intensive women’s labor was, and is, the consistent driving force behind the development and expansion of agrarian and production economies worldwide. Thus, the accurate characterization of female behavior in prehistory is vital for the complete understanding of human adaptive strategies and long-term cultural change.

## MATERIALS AND METHODS

### Experimental design

The study aims were twofold: (i) to generate a comparative data set of CSG properties from living women of known behavior with which to interpret prehistoric female CSG and (ii) to use these comparative data to better understand the sex differences in mobility patterns and temporal change that accompanied the intensification of agriculture in Central Europe. We hypothesized that the observed lower mean bone strength and less pronounced trends through time among prehistoric agricultural women relative to men in this region were an oversimplification of female behavior resulting from an inadequate understanding of biological differences in the bony response to loading. Thus, we aimed to address this through comparison to living women to better elucidate female behavioral complexity through agricultural intensification in Central Europe.

Sports for inclusion in the study were selected on the basis of the specific patterns of limb loading generated: (i) endurance running, low upper limb loading and high, unidirectional lower limb loading; (ii) football (soccer), low upper limb loading and high, multidirectional lower limb loading; and (iii) rowing, moderate unidirectional upper and lower limb loading. Athletes from these sports as well as an additional group of healthy, recreationally active control subjects were recruited (see details below). All participants were healthy adults, predominantly of European descent living in the United Kingdom, and all were between the ages of 19 and 43 years. The following exclusion criteria were established prior to recruitment and were applied to both athletes and control subjects: any medical condition or medication known to interfere with bone metabolism, any current pregnancy, 18 years of age or younger, or peri- or postmenopausal status. Additional exclusion criteria for athletes were participation in the sport of interest for fewer than 3 years, any significant injury within the past year that rendered them inactive for over 1 month, or any current intensive participation in another sport other than the one for which they were recruited. Additional exclusion criteria for control subjects were any current or past participation in competitive sport and any current or past participation of more than 3 hours a week of weight-bearing intensive physical activity.

All participants were recruited through the Cambridge University Women’s Boat Club, Women’s Association Football Club, Athletics Club, Hare and Hounds, and Triathlon Club, as well as the Cambridge and Coleridge Athletics Club, the Cambridge Triathlon Club, the Beyond the Ultimate Jungle Ultra 2016, the Everest Trail Race 2016, several University of Cambridge colleges, and the Graduate Union. Participants filled out a health and activity history questionnaire to determine their athletic training history, recreational physical activity, medical and injury history, and menstrual history (see the Supplementary Materials). The studies of varsity female athletes and ultramarathon runners were approved by the Cambridge University Human Biology Research Ethics Board (HBREC.2015.25 and HBREC.2016.14), and ethical approval for the use of pQCT was obtained from the National Health Service (NHS) Health Research Authority National Research Ethics Service (NRES) Committee East of England–Cambridge East (15/EE/0017). All living participants provided written informed consent prior to their participation in the study. Descriptive statistics for all living women included in the study are available in table S1. See the Supplementary Materials for further details on recruitment and sport groups. Target sample sizes for living women were based on the sample sizes of prehistoric women (see below) to ensure comparability and range from 11 among football players to 37 among controls. Sample sizes among athletes were limited most by the availability of women who fit the appropriate criteria for study inclusion, particularly with regard to sport and loading history. Three rounds of recruitment were performed to maximize the athlete sample sizes.

The prehistoric skeletal sample consisted of females from Central/Southeast European agricultural populations (see fig. S1), spanning portions of four time periods: the Neolithic (Early; ~5300 to 4600 cal BC), Bronze Age (Early and Middle; ~2300 to 1450 BC), Iron Age (Early through Late; ~850 BC to 100 AD), and Medieval (Early; ~800 to 850 AD). Details on all prehistoric cemeteries included in the analyses are available in table S2. Age and sex estimates were determined according to the methods outlined by Buikstra and Ubelaker (*58*), and only skeletally mature adults with fully fused epiphyses were included. Sample sizes for prehistoric material were limited by the availability of skeletal remains of appropriate age, time period, and preservation and range from 11 to 34 depending on the skeletal element and property being examined. Sample sizes were lower for interlimb strength proportions due to the necessity for both a well-preserved humerus and tibia from the same skeleton and range from 6 to 28 individuals.

### Quantification of CSG properties and shape indices

The CSG properties of interest in this study were the polar second moment of area (*J*), a measure of torsional and twice average bending rigidity in two perpendicular planes (in this case, the maximum and minimum axes), and the shape ratio *I*_max_/*I*_min_, a measure of the distribution of bone about these major and minor axes (*59*).

Data from prehistoric skeletal remains were collected using either 3D laser surface scanning (tibiae) or silicone molding (humeri). For tibiae, a 3D model of the complete bone was obtained with a portable a NextEngine desktop laser scanner. Only the best-preserved tibia for each individual was included, but if both elements were equally well preserved, then preference was given to the right side. In addition to individual scan surfaces of the proximal and distal joints, 3D models were composed of 10 individual scan surfaces taken during a 360° rotation. Scans were taken using the HD quality setting in ScanStudio HD Pro (version 1.3.2). The 3D models were trimmed, aligned, and fused using ScanStudio HD Pro and Rapidform XOR. Tibiae were oriented through the alignment of the *x*, *y*, and *z* axes to anatomical planes following the definitions provided by Ruff (*60*). Further details of the laser scanning procedure are reported by Davies and colleagues (*61*). Cross-sectional geometric properties were calculated from finished 3D tibial models at 50% of maximum bone length (parallel to the long axis of the diaphysis) using custom-built AsciiSection software (*61*). This software calculates CSG properties and shape indices for the periosteal contour alone; all CSG properties analyzed in this study refer to these “solid” section properties unless otherwise specified.

The left and right humeral CSG properties were obtained using a silicone molding method (*62*). Periosteal silicone molds were taken at 35% of the maximum length of the left and/or right humeri using Coltène President polyvinyl siloxane putty. Molds were then scanned in anatomical orientation on a flatbed document scanner, oriented with the *x* axis mediolaterally and the *y* axis anteroposteriorly. The resulting mold images were imported into Adobe Photoshop, where the periosteal contour was traced, resulting in a solid cross-sectional image. The cross-sectional images were then imported into ImageJ (http://rsbweb.nih.gov/ij/), and the solid CSG properties were quantified from them using BoneJ, a bone image analysis plug-in (*63*). Solid-section CSG properties have been shown to correspond strongly with true CSG properties derived from both the periosteal and endosteal contours across large regions of the diaphysis (*51*).

Data from living subjects were collected using pQCT (XCT-3000; Stratec Medizintechnik GmbH). All testing took place at the Phenotypic Adaptability, Variation and Evolution Imaging and Performance Laboratory in the Department of Archaeology at the University of Cambridge. Body weight was recorded in kilograms with a SECA electronic scale. Maximum humeral and tibial lengths were obtained from participants using sliding calipers. Cross-sectional images were obtained using pQCT at the 35% section location of the left and right humeri and the 50% section location of the right tibia. Cross-sectional pQCT images were imported into ImageJ, and the soft tissue was cropped out prior to the image being thresholded using the Optimise Threshold function. The medullary cavity was then artificially filled using the Fill Holes option. This resulted in solid cross sections from the living women to generate periosteally derived solid CSG property estimates that would be directly comparable to those from the prehistoric women. CSG properties were then quantified from these solid sections using BoneJ.

### Size-standardization and statistical analyses

Both humeral and tibial *J* were standardized to appropriate measures of body size following the method of Ruff (*59*): *J*/[estimated or true body mass × (maximum bone length^2^)]. For prehistoric skeletal remains, body mass was estimated using the equations for European Holocene populations derived by Ruff and colleagues (*64*), from an average of measurements from the left and right lower limb.

All data distributions were checked for normality using the Kolmogorov-Smirnov test, and outliers exceeding three SDs from the mean were removed from analyses. All data were normally distributed with the exception of left humeral *J* among rowers; the Kruskal-Wallis test was used to examine differences in left humeral *J*, with Mann-Whitney two-tailed tests used for post hoc comparisons. In all other instances, one-way ANOVA was used to test for group differences in CSG properties, using Hochberg’s GT2 or Games-Howell post hoc tests. An alpha level of 0.05 or less was considered statistically significant, and two-sided tests were used. CIs for the mean are provided as 95% CI, and all summary statistics are provided as mean (SD) unless otherwise stated. The relative distribution of upper and lower limb loading was assessed using linear regression of mean raw humeral *J* of the upper limbs combined and raw tibial *J* to produce standardized residuals, which were tested for group differences using ANOVA. All statistical analyses were conducted in SPSS version 23. Variability in humeral and tibial CSG properties was evaluated through the calculation of coefficients of variation [(SD/mean) × 100].

## Supplementary Material

http://advances.sciencemag.org/cgi/content/full/3/11/eaao3893/DC1

## SUPPLEMENTARY MATERIALS

Supplementary material for this article is available at http://advances.sciencemag.org/cgi/content/full/3/11/eaao3893/DC1 Supplementary Materials and Methods fig. S1. Map of Central and Southeast Europe indicating the sampled cemeteries in approximate chronological order. table S1. Descriptive statistics of living women. table S2. Prehistoric skeletal sample details. data file S1. Screening questionnaire for athletes. data file S2. Screening questionnaire for control subjects. data file S3. Health and activity questionnaire for athletes. data file S4. Health and activity questionnaire for control subjects. References (*65*–*76*)
