# Supplementary material for: Prehistoric women’s manual labor exceeded that of athletes through the first 5500 years of farming in Central Europe
Source: Sci Adv. 2017 Nov 29;3(11):eaao3893. doi: 10.1126/sciadv.aao3893 (PMC5710185; doi:10.1126/sciadv.aao3893)
Supplement: http://advances.sciencemag.org/cgi/content/full/3/11/eaao3893/DC1 [file supp_3_11_eaao3893__index.html]

Science Advances | Science Advances

## Supplementary Materials

**This PDF file includes:**

- Supplementary Materials and Methods
- fig. S1. Map of Central and Southeast Europe indicating the sampled cemeteries in approximate chronological order.
- table S1. Descriptive statistics of living women.
- table S2. Prehistoric skeletal sample details.
- data file S1. Screening questionnaire for athletes.
- data file S2. Screening questionnaire for control subjects.
- data file S3. Health and activity questionnaire for athletes.
- data file S4. Health and activity questionnaire for control subjects.
- References (*65–76*)

Download PDF

**Files in this Data Supplement:**

- Adobe PDF - aao3893\_SM.pdf
