# Supplementary material for: Prehistoric women’s manual labor exceeded that of athletes through the first 5500 years of farming in Central Europe
Source: Sci Adv. 2017 Nov 29;3(11):eaao3893. doi: 10.1126/sciadv.aao3893 (PMC5710185; doi:10.1126/sciadv.aao3893)
Supplement: http://advances.sciencemag.org/cgi/content/full/3/11/eaao3893/DC1 [file aao3893_SM.pdf]

Science Advances

AAAS

advances.sciencemag.org/cgi/content/full/3/11/eaao3893/DC1

Supplementary Materials for

Prehistoric women's manual labor exceeded that of athletes through the first 5500 years of farming in Central Europe

Alison A. Macintosh, Ron Pinhasi, Jay T. Stock

Published 29 November 2017, *Sci. Adv.* **3**, eaao3893 (2017)
DOI: 10.1126/sciadv.aao3893

This PDF file includes:

- Supplementary Materials and Methods
- fig. S1. Map of Central and Southeast Europe indicating the sampled cemeteries in approximate chronological order.
- table S1. Descriptive statistics of living women.
- table S2. Prehistoric skeletal sample details.
- data file S1. Screening questionnaire for athletes.
- data file S2. Screening questionnaire for control subjects.
- data file S3. Health and activity questionnaire for athletes.
- data file S4. Health and activity questionnaire for control subjects.
- References (*65–76*)

Supplementary Materials

Supplementary Materials and Methods

Rowers were recruited through the Cambridge University Women's Boat Club, and data from eighteen women were included in analyses. These participants ranged in age from 19-28 years. At the time of data collection, all rowers were currently participating in sweep-style rowing, in which each athlete has only one oar, rowing either bow-side (oar to the athlete's left side) or stroke-side (oar to the athlete's right side). The sample consists of relatively even numbers on each side: six stroke-side rowers, eight bow-side rowers, and four athletes who row interchangeably. In addition to sweep rowing, many of the athletes have previously, or currently still do, also participate in sculling, in which each rower has two oars. All rowers had been actively training/competing for at least four years (4-13 years), many at an international level, and four of the 18 women began rowing prior to menarche.

All football players were recruited through the Cambridge University Women's Association Football Club, and ranged in age from 19-27 years. Eleven football players were included in the study, and all had been actively training/competing for at least four years (4-18 years), some at an international level. Ten of the eleven women began their sport prior to menarche (up to seven years pre-menarcheal).

Twelve endurance runners were recruited through the Cambridge University Athletics Club, the Cambridge University Hare & Hounds, the Cambridge & Coleridge Athletics Club, the Cambridge University Triathlon Club, and the Cambridge Triathlon Club. These participants ranged in age from 19 through 33 years. Athletes had been actively training/competing for at least 3.5 years at the time of data collection (3.5-16 years), some at the national and international level, and five of the twelve women began their sport prior to menarche (up to three years pre-menarcheal). Data from a further five runners obtained during a different research project were also included in analyses; these five athletes were originally recruited from participants in the Beyond the Ultimate Jungle Ultra 2016 and the Everest Trail Race 2016, both multi-day 230 km foot races. These five athletes ranged in age from 31 through 43 years, and all had been actively training/competing in their sport for at least five years (5-15 years) at the time of data collection. None began training and competing in running prior to menarche.

Control subjects were recruited through several University of Cambridge colleges and through the University of Cambridge Graduate Union, and a total of 37 participants were included in analyses. Control subjects ranged in age from 19-32 years of age.

Supplementary Figures

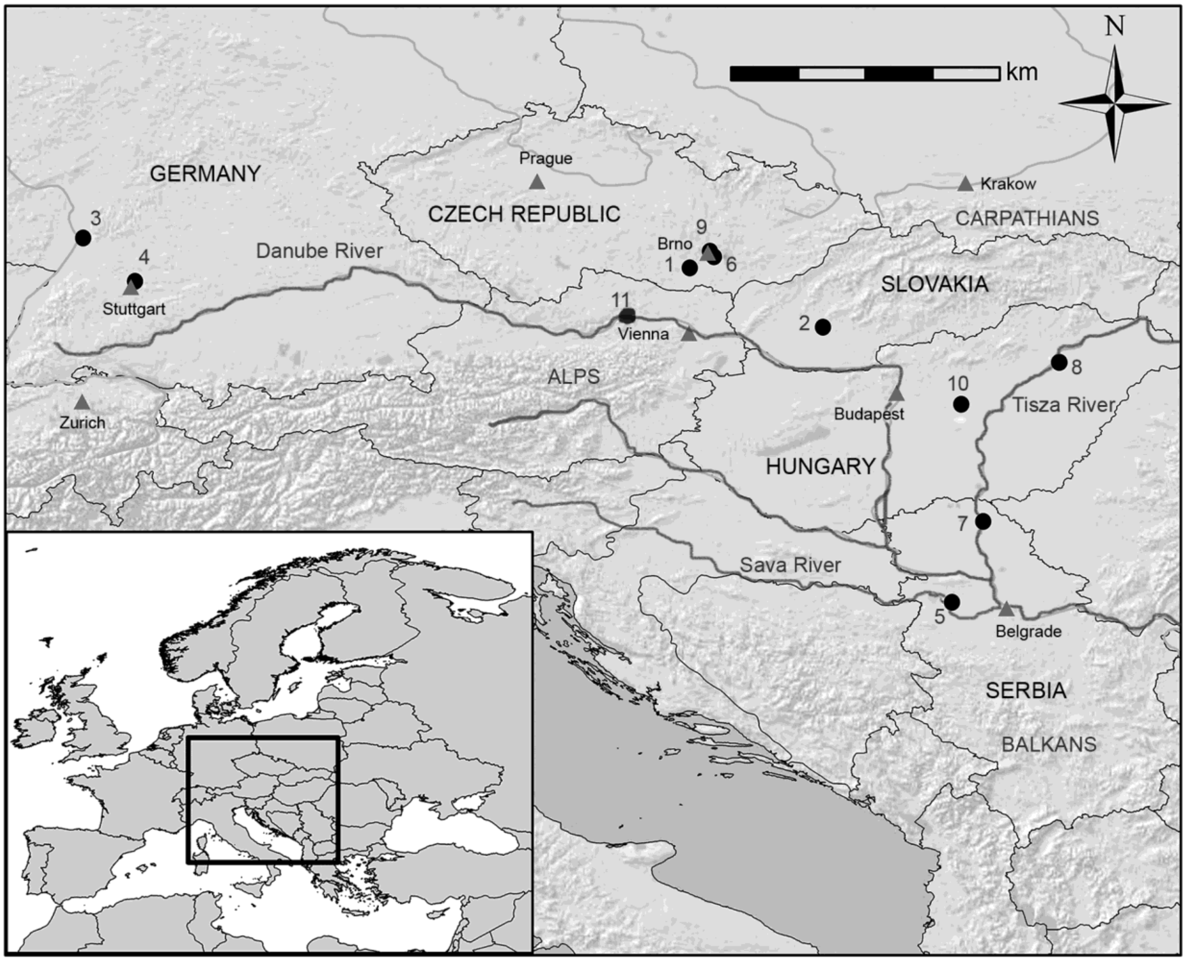

fig. S1. Map of Central and Southeast Europe indicating the sampled cemeteries in approximate chronological order. 1. Vedrovice 2. Nitra Horné Krškany 3. Schwetzingen 4. Stuttgart-Mühlhausen 5. Hrtkovci-Gomolava 6. Brno-Tuřany 7. Ostojićevo 8. Polgár Kenderföld 9. Brno-Maloměřice 10. Tápiószele 11. Pottenbrunn. Reproduced from original by Macintosh and colleagues (9) (http://www.sciencedirect.com/science/article/pii/S0305440314003331).

Supplementary Tables

table S1. Descriptive statistics of living women.

Variable	Endurance runners	Football players	Rowers	Healthy controls
# of individuals	18	11	18	37
Age (yrs)	30 (5.67)	23 (3.52)	23 (2.60)	23 (3.51)
Stature (cms)	167.17 (7.67)	164.37 (4.38)	174.04 (5.88)	167.38 (7.32)
Body mass (kgs)	56.78 (6.07)	63.55 (5.71)	70.71 (9.49)	61.26 (10.88)
Age at menarche (yrs)	13.64 (1.47)	12.82 (1.6)	12.72 (0.96)	12.92 (1.77)
**Training history**				
Starting age (yrs)	17.5 (4.25)	9.0 (2.37)	15.0 (2.98)	-
Current training intensity (hrs/wk)	9.0 (4.34)	6.5 (1.72)	18.0 (2.77)	-
Current kms covered per week	69.5 (24.72)	-	118.0 (30.94)	-
Sport-specific training (yrs)	10.1 (4.55)	12.5 (5.03)	7.0 (2.35)	-
Training relative to age at menarche (yrs)[a]	4.0 (5.18)	-4.1 (2.26)	2.1 (3.66)	-

values given as: mean (standard deviation); 'a': timing of sport initiation relative to menarche in years, negative values indicate the number of years prior to menarche that training was initiated, positive values indicate the number of years after menarche before training was initiated

table S2. Prehistoric skeletal sample details.

Time Period and Culture	Approximate Date (BC)*	Cemetery	Cemetery Location
**Neolithic**			
LBK	5300-5100*	Vedrovice	Czech Republic
LBK	5370-4980*	Nitra Horné Krškany	Slovakia
LBK	5260-5010*	Schwetzingen	Germany
LBK	5200-4960*	Stuttgart-Muhlhaüsen	Germany
**Bronze Age**			
Únětice	2200-2000	Brno-Tuřany	Czech Republic
Maros	~1600/1500	Ostojićevo	Serbia
Füzesabony	1550-1450	Polgár Kenderföld	Hungary
**Iron Age**			
Bosut	850-600/500	Hrtkovci-Gomolava	Serbia
Celtic	400-200	Brno-Maloměřice	Czech Republic
Scythian	385-100AD*	Tápiószele	Hungary
**Early Medieval**			
Slavonic	~800-850 AD	Pottenbrunn	Lower Austria

* indicates calibrated radiocarbon date; LBK= *Linearbandkeramik*; approximate dates from (30, 65–76).

Data Files

data file S1. Screening questionnaire for athletes.

Musculoskeletal Adaptation to Habitual Activity

Screening Questionnaire- Athletes

Office use only: Participant Number __ __ __

Date (DD/MM/YY): ………………………

Physical Activity

How many hours per week on average do you currently engage in structured competitive sport-specific training (including weight training)? ……………………………………

Over the past 12 months, have you:

i) been regularly training/competing in your sport? YES ☐ NO ☐

ii) had any injury that has made you unable to train for more than one month? YES ☐ NO ☐

iii) regularly trained/competed in any other sport? YES ☐ NO ☐

If yes, what sport and how many hours per week on average?

……………………………………………………………………………………………………

Health History

1) Have you ever had any of the following conditions known to affect bone?

	YES		YES
Stomach or bowel problems	☐	Arthritis or other chronic inflammatory conditions	☐
Cystic fibrosis	☐		
Thyroid or parathyroid problems	☐	Extended periods of immobility	☐
Type I diabetes	☐	Prolonged steroid use	☐
Chronic liver disease	☐	Eating disorder	☐
Chronic kidney disease	☐	Cancer	☐
Bone fracture in last 3 months	☐	with chemotherapy/radiation	☐

2) If you selected any of the above conditions, or have any other medical condition, please provide details below (condition, dates, etc):

3) Do you have an implanted pacemaker or defibrillator? YES ☐ NO ☐

4) If you are currently taking any medication (including asthma inhalers), vitamins or supplements, hormonal contraception, and/or hormonal replacement therapy, please provide details below (e.g., type of medication, dosage):

5) Are you currently pregnant or breastfeeding, or have you been within the last 12 months? YES ☐ NO ☐

data file S2. Screening questionnaire for control subjects.

Musculoskeletal Adaptation to Habitual Activity

Screening Questionnaire- Healthy Controls

Office use only: Participant Number __ __ __

Date (DD/MM/YY): ………………………

Physical Activity

Have you ever regularly participated in structured competitive sport (at least twice a week intensive sport-specific training and competition)? YES ☐ NO ☐

If yes, please briefly provide details:

Please list any recreational physical activities in which you currently participate on a regular basis (at least once a week):

Activity	Average # of sessions per week	Average duration per session (minutes)

Health History

1) Have you ever had any of the following conditions known to affect bone?

	YES		YES
Stomach or bowel problems	☐	Arthritis or other chronic inflammatory conditions	☐
Cystic fibrosis	☐		
Thyroid or parathyroid problems	☐	Extended periods of immobility	☐
Type I diabetes	☐	Prolonged steroid use	☐
Chronic liver disease	☐	Eating disorder	☐
Chronic kidney disease	☐	Cancer	☐
Bone fracture in last 3 months	☐	with chemotherapy/radiation	☐

2) If you selected any of the above conditions, or have any other medical condition, please provide details below (condition, dates, etc):

3) Do you have an implanted pacemaker or defibrillator? YES ☐ NO ☐

4) If you are currently taking any medication (including asthma inhalers), vitamins or supplements, hormonal contraception, and/or hormonal replacement therapy, please provide details below (e.g., type of medication, dosage):

5) Are you currently pregnant or breastfeeding, or have you been within the last 12 months? YES ☐ NO ☐

data file S3. Health and activity questionnaire for athletes.

Musculoskeletal Adaptation to Habitual Activities in Women: Health and Activity Questionnaire

Athletes

Office Use Only: Participant Number __ __ __

SECTION A: CURRENT SPORT-SPECIFIC TRAINING

1. In which sport do you currently participate?
Rugby ☐ Rowing ☐ Equestrian ☐ Endurance running ☐

a) if rowing, on which side do you row? Stroke ☐ Bow ☐

2. Over the past year (12 months), how many hours per week (on average) have you spent training/competing in this sport? (not including weight-training)

a) In season (hrs/week, # of sessions/week) …………………………………………

b) Off season (hrs/week, # of sessions/week) …………………………………………

3. **For endurance runners only**: What is the average number of kilometers you run during training/competition in a typical week? ……………………………

4. **For rowers only**: What is the average number of kilometers you row during training/competition in a typical week? (*including erging*) …………………

5. Over the past year, how many hours per week (on average) have you spent engaging in weight-training?

a) In season (hrs/week and # of sessions/week) ………………………………………

b) Off season (hrs/week and # of sessions/week) ……………………………………

6. Please list any other **recreational** physical activity (not structured competitive sport) that you currently engage in on a regular basis (at least once a week for two months or more). Examples: jogging, cycling, lane swimming, pilates, yoga, etc.

**Activity**	**# of hours/week**	**# of sessions/week**

<u>SECTION B</u>:
<u>PAST ATHLETIC HISTORY</u>

***We are interested in the effects of physical activity performed both before and after puberty, so to the best of your ability please answer the following question:**

How old were you when you had your first period (years?) …………………
Date if known (YYYY or MM/YYYY) ………………………………

1. How old were you when you began actively training for your sport (at least 2 sessions per week)?
Years of age ………… Approximate date if possible (MM/YYYY) ……………

2. For how many years total have you actively participated in structured training and competition for your sport? …………………………

3. How old were you when you first began weight-training, if applicable?

Years of age ………… Approximate date if possible (MM/YYYY) ……………

4. What is the average number of hours per week you spent training for your sport (not including weight-training):

a) <u>*up to and including*</u> the year of your first period? …………………………………

b) the year <u>*after*</u> your first period until present? ………………………………………

5. How many hours per week on average did you weight-train (if applicable):

a) <u>*up to and including*</u> the year of your first period? …………………………………

b) from the year <u>*after*</u> your first period until present? …………………………………

6. Please list any **<u>recreational</u>** physical activity (not structured competitive sport) that you engaged in on a regular basis (at least once a week for two months or more). Examples: lane swimming, cycling, jogging, yoga, farm work

a) <u>*up to and including*</u> the year of your first period?

**Activity**	**Ages (yrs)**	**# of hours/week**	**# of sessions/week**
e.g., ballet	4-8	1	1

b) from the year *after* your first period until present?

Activity	Ages (yrs)	# of hours/week	# of sessions/week
e.g., lane swimming	21-present	1	1

7. Which of the following best describes your activity level from the ages of **8-13 years old**:
   Competitive in sport ☐ Exercising for recreation ☐ Mostly sedentary ☐

8. Which of the following best describes your activity level from the ages of **14-17 years old**:
   Competitive in sport ☐ Exercising for recreation ☐ Mostly sedentary ☐

9. Which of the following best describes your activity level from the ages of **18 to present**:
   Competitive in sport ☐ Exercising for recreation ☐ Mostly sedentary ☐

10. Please list all sports you have ever played at a structured **competitive level** (non-recreational) for more than six months consistently. You do not need to include the sport for which you have been recruited to this study.

Sport	Age(s) Played	Highest Level Played	Position
e.g. volleyball	10-11, 15-16	school	setter
e.g. hockey	18-20	national	forward

SECTION C: FRACTURE AND INJURY HISTORY

1. If you have ever had a stress fracture, please provide details below.

	Bone	Age (yrs)	Time missed & Dates
e.g.	fibula	15	12 weeks, Feb - April 2002

2. Other than stress fractures, if you have ever broken any bones, please provide details below.

	Bone	Age (yrs)	Cause	Time missed & dates
e.g.	radius	21	rugby tackle	6 weeks, May - June 2011

3. If you have ever had any other injury that affected your ability to exercise for more than 1 month, please provide details below. (Examples: tendonitis, torn muscle, sprained ankle etc.)

	Injury	Age (yrs)	Time missed & dates
e.g.	torn hamstring	19	8 weeks, Sept - Nov 2008

4. If you have ever had to stay in a wheelchair or bed for more than 4 weeks, please provide details below.

	Cause	Age (yrs)	Duration and dates
e.g.	car accident	22	6 weeks, Dec - Feb 2010

SECTION D:
MEDICAL HISTORY

1. What was your birth weight? ………………………………

2. Were you born prematurely (<35 weeks gestation)? YES ☐ NO ☐ DON'T KNOW ☐
   If yes, what was your gestational age (weeks)? ………………………

3. If you have ever had a serious medical condition or illness, please give details below:

4. Has any blood relative ever suffered from osteoporosis? YES ☐ NO ☐

5. Do you actively avoid dairy products? YES ☐ NO ☐
   If yes, do you take calcium and/or vitamin D supplementation? YES ☐ NO ☐

6. Have you ever used hormonal contraceptives at any point in your life? YES ☐ NO ☐
   If yes, do you currently use them? YES ☐ NO ☐

7. How old were you when you first started taking hormonal contraceptives (years)? ………
   Please provide approximate date if possible (MM/YYYY). …………………………

8. Please provide as many of the following details as possible about your current and past hormonal contraceptive use.

**Contraceptive Name**	**Contraceptive Type**	**Age(s) when taken (years)**
e.g. Yaz	Oral	16-24 years old

9. If you ever consume alcoholic drinks, please indicate how many units of alcohol you consume on average per week? ……………………………

(1 unit= 1/2 pint of beer/lager, 1 glass of wine, 1 pub measure of spirits)

10. If you have ever smoked cigarettes or other tobacco products, please provide the following information:

a) Do you currently smoke? YES ☐ NO ☐
b) How old were you when you started smoking (years)? ……………
c) For how many years have you, or did you, smoke? …………………
d) How many cigarettes/tobacco products do you, or did you, smoke per day on average? …………………………………………

SECTION E: MENSTRUAL HISTORY

1. Have you been pregnant, given birth, or breastfed in the past 12 months? YES ☐ NO ☐

2. If you have children, what were their birth weights? ………………………………………

3. Do you currently have a regular menstrual cycle (10-13 periods/year)? YES ☐ NO ☐

If no, how many periods have you had in the last year (12 months)? ……………

4. What was the date of the *first day* of your most recent period (if known)? ………………

5. Have you always had a regular menstrual cycle (*10-13 periods/year*)? YES ☐ NO ☐

6. Have you ever gone more than 3 months without a period? YES ☐ NO ☐

7. Have you had at least one period in the last 6 months? YES ☐ NO ☐

data file S4. Health and activity questionnaire for control subjects.

Musculoskeletal Adaptation to Habitual Activities in Women: Health and Activity Questionnaire

Healthy Control Subjects

Office Use Only: Participant Number __ __ __

SECTION A: CURRENT PHYSICAL ACTIVITY

1. Please list any regular physical activity in which you currently participate on a regular basis (at least once a week for two months or more).

Examples: jogging, walking, tennis, lifting weights, volleyball, aerobics, pilates, swimming

Activity	# of hours/week	# of sessions/week	Duration/session

SECTION B: PAST PHYSICAL ACTIVITY

***We are interested in the effects of physical activity performed both before and after puberty, so to the best of your ability please answer the following question:**

How old were you when you had your first period (years?) …………………

Date if known (YYYY or MM/YYYY) ………………………………

1. Please list any regular physical activity that you engaged in on a regular basis (at least once a week for two months or more):

a) *up to and including* the year of your first period?

Activity	Ages (yrs)	# of hours/week	# of sessions/week
e.g., ballet	4-8	1	1

b) from the year *after* your first period until present?

**Activity**	**Ages (yrs)**	**# of hours/week**	**# of sessions/week**
e.g., jogging	18-present	1-2	2

2. Which of the following best describes your activity level from the ages of **8-13 years old**:
Competitive in sport ☐ Exercising for recreation ☐ Mostly sedentary ☐

3. Which of the following best describes your activity level from the ages of **14-17 years old**:
Competitive in sport ☐ Exercising for recreation ☐ Mostly sedentary ☐

4. Which of the following best describes your activity level from the ages of **18 to present**:
Competitive in sport ☐ Exercising for recreation ☐ Mostly sedentary ☐

SECTION C:
FRACTURE AND INJURY HISTORY

1. If you have ever had a stress fracture, please provide details below.

	**Bone**	**Age (yrs)**	**Time missed & Dates**
e.g.	fibula	15	12 weeks, Feb - April 2002

2. Other than stress fractures, if you have ever broken any bones, please provide details below.

	**Bone**	**Age (yrs)**	**Cause**	**Time missed & dates**
e.g.	radius	21	rugby tackle	6 weeks, May - June 2011

3. If you have ever had any other injury that affected your ability to exercise for more than 1 month, please provide details below. (Examples: tendonitis, torn muscle, sprained ankle etc.)

	**Injury**	**Age (yrs)**	**Time missed & dates**
e.g.	torn hamstring	19	8 weeks, Sept - Nov 2008

4. If you have ever had to stay in a wheelchair or bed for more than 4 weeks, please provide details below.

	Cause	Age (yrs)	Duration and dates
e.g.	car accident	22	6 weeks, Dec - Feb 2010

SECTION D:
MEDICAL HISTORY

1. What was your birth weight? ……………………………

2. Were you born prematurely (<35 weeks gestation)? YES ☐ NO ☐ DON'T KNOW ☐
   If yes, what was your gestational age (weeks)? …………………………

3. If you have ever had a serious medical condition or illness, please give details below:

4. Has any blood relative ever suffered from osteoporosis? YES ☐ NO ☐

5. Do you actively avoid dairy products? YES ☐ NO ☐
   If yes, do you take calcium and/or vitamin D supplementation? YES ☐ NO ☐

6. Have you ever used hormonal contraceptives at any point in your life? YES ☐ NO ☐
   If yes, do you currently use them? YES ☐ NO ☐

7. How old were you when you first started taking hormonal contraceptives (years)? ………
   Please provide approximate date if possible (MM/YYYY). …………………………

8. Please provide as many of the following details as possible about your current and past hormonal contraceptive use.

	Contraceptive Name	Contraceptive Type	Age(s) when taken (years)
e.g.	Yaz	Oral	16-24 years old

9. If you ever consume alcoholic drinks, please indicate how many units of alcohol you consume on average per week? ……………………………
   (1 unit= 1/2 pint of beer/lager, 1 glass of wine, 1 pub measure of spirits)

10. If you have ever smoked cigarettes or other tobacco products, please provide the following information:

a) Do you currently smoke? YES ☐ NO ☐
b) How old were you when you started smoking (years)? ……………
c) For how many years have you, or did you, smoke? …………………
d) How many cigarettes/tobacco products do you, or did you, smoke per day on average? …………………………………………

SECTION E:
MENSTRUAL HISTORY

1. Have you been pregnant, given birth, or breastfed in the past 12 months? YES ☐ NO ☐

2. If you have children, what were their birth weights? ………………………………………

3. Do you currently have a regular menstrual cycle (10-13 periods/year)? YES ☐ NO ☐
If no, how many periods have you had in the last year (12 months)? ……………

4. What was the date of the *first day* of your most recent period (if known)? ………………

5. Have you always had a regular menstrual cycle (*10-13 periods/year*)? YES ☐ NO ☐

6. Have you ever gone more than 3 months without a period? YES ☐ NO ☐

7. Have you had at least one period in the last 6 months? YES ☐ NO ☐
